# Characteristics and outcomes analysis of ovarian Sertoli–Leydig cell tumors (SLCTs): analysis of 15 patients

**DOI:** 10.1186/s13048-021-00909-7

**Published:** 2021-11-04

**Authors:** Guangning Wang, Ran Zhang, Chuan Li, Aiping Chen

**Affiliations:** 1 Department of Gynecology and Obstetrics, The Affiliated Hospital of Qingdao University, NO.16 Jiangsu Road, Qingdao, 266000 Shandong China; 2 Qingdao University, Qingdao, China

**Keywords:** Sertoli–Leydig cell tumors, SLCTs, Conservative surgery, Chemotherapy

## Abstract

**Introduction:**

Because of limited information of Sertoli–Leydig cell tumors (SLCTs), the objective aimed to describe clinical parameters, management and treatment results of SLCTs.

**Material and methods:**

We retrospectively reviewed 15 cases with SLCTs, who were treated in the Affiliated Hospital of Qingdao University between 2009 and 2020. Data of clinical parameters and treatment was studied.

**Results:**

The age ranged 25–69 years. Elevated testosterone was observed in 4 patients. FIGO-stage: 14 were at Ia(10 moderately differentiated, 3 poorly differentiated, 5 retiform pattern).1 was at Ic. Patients with retiform pattern were more likely to exhibit endocrine function (*p* = 0.019, w = 0.61) and tumor diameter was significantly bigger in no endocrine function (*p* = 0.012, d = 1.52). All patients received surgical treatment. 8 received postoperative chemotherapy. The median follow-up was 66 months (20–112 months). 1 patient relapsed within 36 months and received cytoreductive surgery. She survived without disease after recurrence treatment. Of 5 patients who performed fertility sparing surgeries with the desire of childbirth, 3 had full-term pregnancy and 1 experienced a miscarriage. Another one has not tried to conceive.

**Conclusion:**

The prognosis of SLCTs is good. Our data showed patients with retiform pattern were more likely to exhibit endocrine function. The diameter of tumor was significantly bigger in no endocrine function. Conservative surgery is the preferred option for patients with the desire of fertility at stage Ia. Postoperative chemotherapy is advised to cases with high-risk factors, but the most effective chemotherapy regimen is still uncertain.

## Introdution

Sertoli-Leydig cell tumors (SLCTs) are derived from ovarian sex-cord stromal neoplasms. These tumors constitute 0.2–0.5% of all ovarian cancer. SLCTs occur over a very wide age (from 1 to 84 years), and the average was 25 [1–3]. It is worth noting that about one-third of SLCTs presented symptoms of androgen excess including hirsutism, acne, and oligomenorrhea-amenorrhe. Occasionally, SLCTs presented with estrogenic manifestations. Excessive estrogen may result in endometrial carcinoma. The other 50% of cases are not functionally active [4, 5]. According to the 2014 WHO histological classification of female reproductive tumors, SLCTs are classified into four types: well-differentiated forms, intermediate differentiation forms (moderately differentiated), poorly differentiated, and retiform.

Because of the rarity of SLCTs, the clinical features and management protocol guidelines remain uncertain. Therefore, the objective of study aimed to evaluate the clinical characteristics and outcome of ovarian SLCTs managed at a single institution to improve the knowledge of SLCTs for oncologists.

## Materials and methods

Fifteen patients with the diagnosis of SLCTs in the Affiliated Hospital of Qingdao University from 2009 to 2020 were reviewed. The clinical data was shown in Table 1. Surgical resection of primary tumor was the initial treatment for all cases. The histology of the primary tumor was reviewed and assessed by two senior pathologists from the Affiliated Hospital of Qingdao University. Tumor were staged according to 2014 International Federation of Gynecology and Obstetrics (FIGO) staging system [6]. Fifteen patients received the initial treatment in our institution and had follow-ups until 2020. In order to compare the pathological features between different endocrine function groups, the categorical data was analyzed by Fisher’s exact test. *p* < 0.05 was considered statistically significant. Institutional review board has approved this study.

**Table 1:** Patient characteristics

No	Age	Clinical manifestation	Parturition	Laboratory	Ultrasound	Histopathology				Treatment		Relapse	Follow-up	
						Side	D (cm)	Diagnosis	Stage	Surgery	Chemotherapy		Period	Status
1	26	Amenorrhea	NO	Testosterone↑ AFP ↑	CS	R	8.5	Moderately differentiated	Ia	USO	–	No	93	DFS + P
2	39	Amenorrhea voice raucity	Yes	Testosterone↑	CS	L	3	Poorly differentiated + R	Ia	USO	NVB×6	No	112	DFS
3	60	Pelvic mass	Yes	Normal	S	L	10	Moderately differentiated	Ia	BSO	–	no	102	DFS
4	28	Pelvic mass	No	CA125 ↑ AFP ↑	CS	L	7	Poorly differentiated +R	Ic	CYS + USO	BEP×4	No	64	DFS
5	60	Postmenopausal bleeding	Yes	CA199 ↑	CS	L	2	Moderately differentiated +atypical	Ia	THBSO	–	No	85	DFS
6	25	Menstrual irregularity	No	Testosterone ↑ AFP ↑	C	L	4	Moderately differentiated + R	Ia	USO	BEP×4	NO	66	DFS + P
7	61	Abdominal pain	Yes	Normal	CS	R	4	Moderately differentiated	Ia	THBSO	–	No	36	DFS
8	48	Abdominal pain	Yes	Normal	CS	R	9	Poorly differentiated	Ia	THBSO+S	BEP×6	No	43	DFS
9	44	Pelvic mass	Yes	Normal	S	R	7.5	Moderately differentiated	Ia	THBSO+S	–	No	49	DFS
10	69	Abdominal pain	Yes	Normal	S	R	5	Moderately differentiated	Ia	THBSO	–	No	20	DFS
11	58	Pelvic mass	Yes	Normal	C	R	10	Moderately differentiated	Ia	THBSO	–	NO	28	DFS
12	65	Postmenopausal bleeding	Yes	CA125↑ CEA↑ AFP↑ CA199↑	–	R	0.5	Poorly differentiated +EC + R	Ia	THBSO+S	TC×6	No	68	DFS
13	26	Amenorrhea	No	Normal	C	L	2.5	Moderately differentiated + R	Ic	USO	BEP×4	YES	87	AWD
14	27	Menstrual irregularity	No	Testosterone↑ AFP	C	L	4	Moderately differentiated + R	Ia	USO	BEP×4	NO	32	DFS + P
15	28	Menstrual irregularity	Yes	Normal	CS	L	6	Moderately differentiated + R	Ia	USO	BEP×4	No	86	DFS

*CS* cyst and solid, *C* cyst, *S* solid, *R* right, *L* left, *EC* endometrial cancer, *USO* unilateral salpingo-oophorectomy, *BSO* bilateral salpingo-oophorectomy, *CYS* cystectomy, *THBSO* total hysterectomy and bilateral salpingo-oophorectomy, *S(treatment)* staging surgery (omentectomy ± pelvic lymphadenectomy), *BEP* (bleomycin + etoposide + cisplatin), *NVB* (nedaplatin + vincristine + bleomycin), *TC* (paclitaxel + carboplatin), *DFS* disease-free survival, *DFS + P* disease-free survival and pregnancy, *AWD* alive without disease after relapse treatment

## Results

### Clinical characteristics

From 2009 to 2020, 15 patients with SLCTs were included in this analysis. The median age was 44 years (25–69 years, Table 1). Six (40%) cases were post-menopausal. And 6 (40%) patients were younger than 30 years.

8 (53.3%) patients presented endocrine function: 3 presented androgenic manifestations (3 had amenorrhea, 1 showed voice raucity), 3 presented menstrual irregularity, 2 presented post-menopausal hemorrhage. 7 (46.7%) patients had no endocrine symptoms but underwent ultrasonic exams due to abdominal pain (*n* = 3) or pelvic mass (*n* = 4). Serum sex hormone level was measured in 14 patients (expect 1 patient diagnosed with endometrial carcinoma) before the operation. Elevated testosterone was observed in 4 patients (2 androgenic manifestations, 2 menstrual irregularity). We re-test their serum testosterone after the surgery. And all were within normal level. Tumor markers was measured in 15 patients. A significant elevation of alpha-fetoprotein (AFP) was observed in 5 patients. Only 2 patients had elevated cancer antigen 125 (CA125).

### Ultrasonographic characteristics

When reviewing the ultrasonographic images, we observed the features were classified into three types: solid-cystic (*n* = 7), solid (*n* = 3) and cystic (*n* = 4). All of 14 patients had a single tumor while 8 (53.3%) located in the left ovary, 6 (40%) located in the right ovary. As for the 1 undetected tumors, the size(d = 0.5 cm)was too small to be not detected by ultrasound. There weren’t papillary projections in tumor.

### Histopathology

**Fig. 1:**
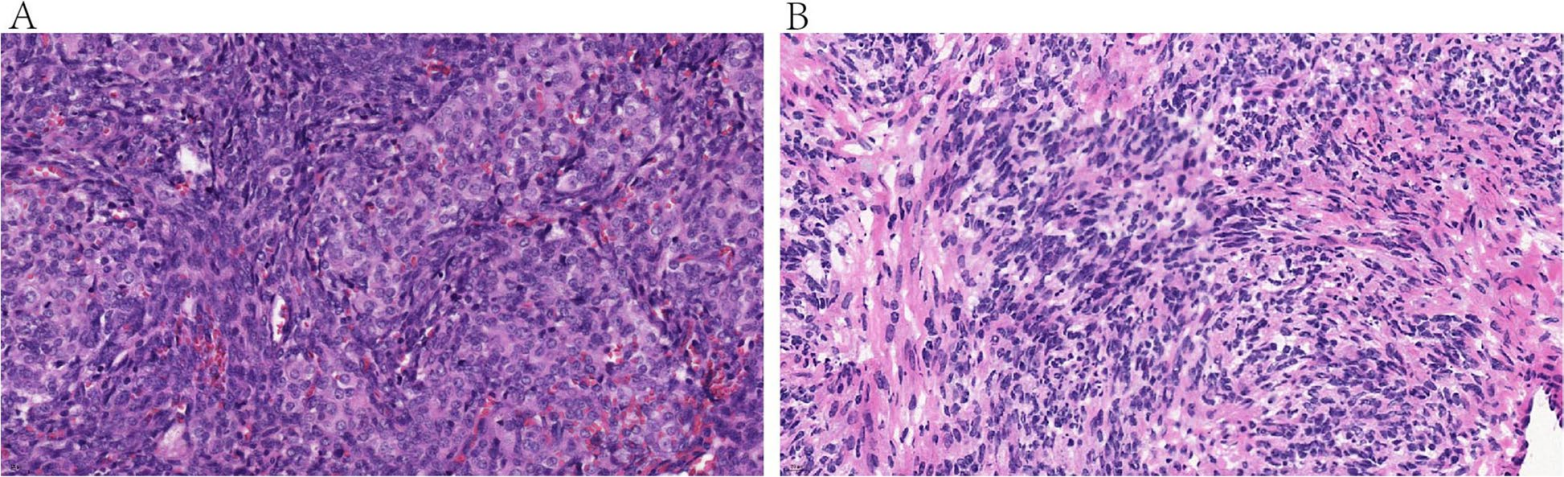
Histopathological images of SLCTs. A Moderately differentiated tumor with immature Sertoli form tubules and fused nests. Several Leydig cell clusters are also seen. B Poorly differentiated tumor with diffuse sheets of immature Sertoli cells and few Leydig cells

According to FIGO system, 15 cases were at stage I, including Ia (*n* = 14) and Ic (*n* = 1). 11 tumors were moderately differentiated (Fig. 1A). 4 were poorly differentiated (Fig. 1B). A retiform pattern was observed in 7 patients of moderate (*n* = 4) or poor (*n* = 3) differentiation. The median size of 15 tumors was 5.0 cm (range 0.5–10.0 cm). Based on the endocrine function, the patients were divided into two group. Patients with retiform pattern were more likely to exhibit endocrine function (*p* = 0.019, w = 0.61; Table 2), but tumor diameter was significantly bigger in no endocrine function group (*p* = 0.012, d = 1.52; Table 2). However, the mean age showed no significant difference (*p* = 0.070, d = 1.02; Table 2).

**Table 2:** Details of patients (endocrine function group vs. no endocrine function group)

Endocrine functions	No. of patients	Mean age (years)	Diameter (cm)	Retiform
YES	8	37.00 ± 16.41	3.81 ± 2.49	6
NO	7	53.57 ± 12.69	7.50 ± 2.36	1
P		0.070(> 0.05)	0.012(< 0.05)	0.019 (< 0.05)

### Therapeutic procedures

Fifteen patients received surgery at the initial treatment. 7 recieved Unilateral salpingo-oophorectomy, 1 recieved bilateral salpingo-oophorectomy, 7 recieved total hysterectomy and bilateral salpingo-oophorectomy and three of them underwent additional standard staging surgery. Among the 8 patients with adjuvant chemotherapy, 6 patients received bleomycin + etoposide + cisplatin (BEP) chemotherapy with 4–6 cycles; 1 patient underwent paclitaxel + carboplatin (TC) regimens with 6 cycles; 1 patient underwent nedaplatin + vincristine + bleomycin (NVB) regimens with 4 cycles.

### Follow-up

Follow up information was available for 15 patients. 14 were alive and exhibited free of disease postoperatively for 20 to 112 months postoperatively. Only one patient had a recurrence. She received Unilateral salpingo-oophorectomy and four cycles BEP chemotherapy at the initial treatment. Thirty-six months later, cytoreductive surgery was performed because of recurrences. She received TC chemotherapy postoperatively. The histology of the second tumor enabled a diagnosis of SLCTs with poorly differentiated. At the last time of follow up, she was alive and exhibited alive without disease after relapse treatment (AWD). Of the 5 patients with the desire to have children, 3 experienced full-term pregnancy and 1 had a miscarriage. Another one has not tried to conceive.

## Discussion

Sex cord-stromal tumors constitute 0.2–0.5% of ovary cancer. Due to the low incidence, limited information of SLCTs is available. According to the previous studies, SLCTs may occur at any age (1–82 years old), the average was 25 [1]. Most of the SLCTs are at stage I, with the lesion being confined to unilateral ovary [7]. In our study, median age was 44 years (25–69 years old). All of 15 SLCTs are confined to unilateral ovary.

Ultrasound morphology of the SLCTs may be variable. A study by V N Demidov et al. showed SLCTs were either solid tumors, or multilocular solid which could be identified by ultrasonic exam [8]. Another study analyzed 207 patients diagnosed as SLCTs. Fifty-eight percent of the tumors were solid and cystic, 38% were solid, and only 4% were cystic [2]. In our study, we observed 7 solid-cystic (*n* = 7), 3 solid (*n* = 3), and 4 cystic (*n* = 4). The identification of imaging findings would widen current knowledge of SLCTs and be useful to improve the accuracy of preoperative diagnosis.

It is worth noting that about one-third of SLCTs presented symptoms of androgen excess including hirsutism, acne, seborrhea, and oligomenorrhea-amenorrhea [2]. Occasionally, SLCTs presented with estrogenic manifestations. Excessive estrogen may result in endometrial carcinoma [5, 9].The co-existence of SLCTs and endometrial carcinoma was reported several times [7, 10–12]. Levent Akman reported two cases with endometrial cancer (well differentiated) [12]. In our series, we reported 1 case of a poorly differentiated Sertoli–Leydig cell tumor with endometrial cancer in a 60-year-old woman. However, approximately 50% of SLCT cases don’t have endocrine manifestation [5]. These make the preoperative diagnosis of Sertoli–Leydig cell tumors difficult. Therefore, women who were suspected with SLCTs may help to improve the accuracy of diagnosis by testing the hormone level.

Because of the rarity of SLTCs, there is no standard guideline in therapy of SLCTs. The treatment depends on the tumor stage, age, and the degree of differentiation. Surgical treatment is still the main therapy to treat SLTCs. For stage Ia disease, it is appropriate to prefer fertility sparing surgeries for young patients with the desire of fertility because of the similar recurrence rate no matter which type of surgery is. According to the previous study, the recurrence rate was 8% in fertility-preservation surgery group. And the recurrence rate was 3% in radical surgery group [13]. Analysis of stage Ic was complicated. Advanced stage was identifed as a poor prognostic factor. Gouy et al. reviewed 13 studies and reported that stage Ic has been correlated with high recurrence rate (around 30%) and high mortality, (around 54%) [13]. Thus, we need more information about Ic to define which type of surgery of SLTCs is the best. In this series, of the 5 patients who performed fertility sparing surgeries with the desire to have children, 3 experienced full-term pregnancy and 1 had a miscarriage. Another one is still unmarried. Another thing to consider is the rupture of tumor. The rupture was identified as a poor prognostic factor [2]. When treating patients suspected with SLCTs. We should take necessary method to avoid a rupture.

The discovered prognostic factors are poor, mainly based on case series. The prognostic predictors reported in literature are stage and degree of differentiation [2, 13–15]. A respective MITO study which evaluated the outcome of 21 SLTCs showed: the five-year survival rate was 92.3 and 67% for stage I and advanced stage died of disease respectively [16].

A study of 64 patients showed five-year overall survival was 92% for intermediate or poorly differentiated SLCT. Another study showed for grade 2–3, the 5-year overall survival was 77%, but for grade 1 it was 100% [16]. Gouy et al. reviewed 13 articles and reported that the relapse rate of SLCTs at stage Ia was slightly lower than that of SLCTs at stage Ic (7% vs. 30%) [13]. In our research, 1 patient experience a recurrence. Thirty-six months after initial surgery, cytoreductive surgery was performed due to pelvic recurrences and TC chemotherapy was followed after the surgery. At the last follow up, she was alive and exhibited AWD. There was also evidence that the prognosis of SLCTs with retiform pattern was worse [17, 18]. Our analysis suggested patients with retiform pattern were more likely to exhibit endocrine function. We speculate the endocrine group may have more aggressive biological behaviors. But the information about it is limited. We need more data to Confirm it.

Moreover, the role of postoperative adjuvant chemotherapy of SLCTs remain controversial because of the lack of prospective studies. According to current literature, chemotherapy was advised as a consolidation treatment for cases with moderately /poorly differentiated, stage Ic, retiform pattern, or with heterologous elements after surgery. Although BEP chemotherapy is mostly frequently used as an adjuvant therapy in SLCT, there is an absence of agreement on the best and most favorable regimen for SLCTs. Other chemotherapy regimens included TP paclitaxel plus cisplatin (TP), ifosfamide + etoposide + cisplatin (VIP), cisplatin + vincristine + bleomycin (PVB) and cisplatin + epirubicin + cyclophosphamide (PAC) [5, 19–21]. A study about treatment of sex cord-stromal ovarian tumors showed that the efficacy of TC or paclitaxel alone is very similar to BEP chemotherapy regimen. While its toxicity is less. A study suggested bevacizumab has activity in the treatment of recurrent sex cord-stromal tumors of the ovary, and its toxicity is acceptable [20]. Unfortunately, these studies didn’t include SLCTs. By searching the ClinicalTrials.gov, few ongoing trials are testing the effect of paclitaxel with carboplatin (NCT01042522) in SLCTs treatment. Further trials are requested to determine new therapeutic approaches for SLCTs.

This study has several limitations. One limitation of this study is that the sample size is small due to the rarity of the disease. Secondly, recent studies show DICER1 mutation are associated with pathogenesis and prognosis in ovarian SLCTs. Therefore, it’s important to identify DICER1 mutation for the patient, the family members, and potential offspring [22, 23]. Although we provided detailed information about the DICER1 mutation. The patient has refused to test DICER1 because of some certain reasons. However, the genetic testing of DICER1 should be recommended.

## Conclusion

In conclusion, the prognosis of SLCTs is good. Our data showed patients with retiform pattern were more likely to exhibit endocrine function and the diameter of tumors was significantly bigger in endocrine function. Fertility-sparing surgery is the preferred option for patients with the desire of fertility at stage Ia. For stage Ic, the appropriate type of surgery is still complicated. Postoperative chemotherapy is advised to cases with high-risk factors such as stage Ic, moderately /poorly differentiated, retiform pattern, or heterologous elements. But the most effective chemotherapy regimen is still uncertain.

## Data Availability

The data during the current study are available from the corresponding author on reasonable request.

## References

[1] Lantzsch T, Stoerer S, Lawrenz K, Buchmann J, Strauss HG, Koelbl H (2001). Sertoli-Leydig cell tumor. Arch Gynecol Obstet.

[2] Young RH, Scully RE (1985). Ovarian Sertoli-Leydig cell tumors. A clinicopathological analysis of 207 cases. Am J Surg Pathol.

[3] Young RH (2005). Sex cord-stromal tumors of the ovary and testis: their similarities and differences with consideration of selected problems. Mod Pathol.

[4] Schneider DT, Orbach D, Cecchetto G, Stachowicz-Stencel T, Brummel B, Brecht IB (2015). Ovarian Sertoli Leydig cell tumours in children and adolescents: an analysis of the European cooperative study group on pediatric rare tumors (EXPeRT). Eur J Cancer.

[5] Gui T, Cao D, Shen K, Yang J, Zhang Y, Yu Q (2012). A clinicopathological analysis of 40 cases of ovarian Sertoli-Leydig cell tumors. Gynecol Oncol.

[6] Prat J (2014). Staging classification for cancer of the ovary, fallopian tube, and peritoneum. Int J Gynaecol Obstet.

[7] Bhat RA, Lim YK, Chia YN, Yam KL (2013). Sertoli-Leydig cell tumor of the ovary: analysis of a single institution database. J Obstet Gynaecol Res.

[8] Demidov VN, Lipatenkova J, Vikhareva O, Van Holsbeke C, Timmerman D, Valentin L (2008). Imaging of gynecological disease (2): clinical and ultrasound characteristics of Sertoli cell tumors, Sertoli-Leydig cell tumors and Leydig cell tumors. Ultrasound Obstet Gynecol.

[9] Weng CS, Chen MY, Wang TY, Tsai HW, Hung YC, Yu KJ (2013). Sertoli-Leydig cell tumors of the ovary: a Taiwanese gynecologic oncology group study. Taiwan J Obstet Gynecol.

[10] Xiao H, Li B, Zuo J, Feng X, Li X, Zhang R (2013). Ovarian Sertoli-Leydig cell tumor: a report of seven cases and a review of the literature. Gynecol Endocrinol.

[11] Melero Cortés LM, Martínez Maestre M, Vieites Pérez-Quintela MB, Gambadauro P (2017). Ovarian Sertoli-Leydig cell tumours: how typical is their typical presentation?. J Obstet Gynaecol.

[12] Akman L, Ertas IE, Gokcu M, Terek MC, Sanci M, Sanli UA (2016). Ovarian sertoli-leydig cell tumors: a multicenter long-term clinicopathological analysis of 27 patients. J Cancer Res Ther.

[13] Gouy S, Arfi A, Maulard A, Pautier P, Bentivegna E, Leary A (2019). Results from a monocentric long-term analysis of 23 patients with ovarian Sertoli-Leydig cell tumors. Oncologist.

[14] Colombo N, Peiretti M, Garbi A, Carinelli S, Marini C, Sessa C (2012). Non-epithelial ovarian cancer: ESMO Clinical Practice Guidelines for diagnosis, treatment and follow-up. Ann Oncol.

[15] Guo Y, Wang J, Li Y, Wang Y (2020). Ovarian Sertoli-Leydig cell tumors: an analysis of 13 cases. Arch Gynecol Obstet.

[16] Sigismondi C, Gadducci A, Lorusso D, Candiani M, Breda E, Raspagliesi F (2012). Ovarian Sertoli-Leydig cell tumors. A retrospective MITO study. Gynecol Oncol.

[17] Kawatra V, Mandal S, Khurana N, Aggarwal SK (2009). Retiform pattern of Sertoli-Leydig cell tumor of the ovary in a 4-year-old girl. J Obstet Gynaecol Res.

[18] Mooney EE, Nogales FF, Bergeron C, Tavassoli FA (2002). Retiform Sertoli-Leydig cell tumours: clinical, morphological and immunohistochemical findings. Histopathology.

[19] Nam SM, Kim JW, Eoh KJ, Kim HM, Lee JY, Nam EJ (2017). A novel clinicopathological analysis of early stage ovarian Sertoli-Leydig cell tumors at a single institution. Obstet Gynecol Sci.

[20] Brown J, Shvartsman HS, Deavers MT, Ramondetta LM, Burke TW, Munsell MF (2005). The activity of taxanes compared with bleomycin, etoposide, and cisplatin in the treatment of sex cord-stromal ovarian tumors. Gynecol Oncol.

[21] Brown J, Brady WE, Schink J, Van Le L, Leitao M, Yamada SD (2014). Efficacy and safety of bevacizumab in recurrent sex cord-stromal ovarian tumors: results of a phase 2 trial of the gynecologic oncology group. Cancer.

[22] de Kock L, Terzic T, McCluggage WG, Stewart CJR, Shaw P, Foulkes WD (2017). DICER1 mutations are consistently present in moderately and poorly differentiated Sertoli-Leydig cell tumors. Am J Surg Pathol.

[23] Karnezis AN, Wang Y, Keul J, Tessier-Cloutier B, Magrill J, Kommoss S (2019). DICER1 and FOXL2 mutation status correlates with Clinicopathologic features in ovarian Sertoli-Leydig cell tumors. Am J Surg Pathol.

